# *JAZF1/SUZ12* gene fusion in endometrial stromal sarcomas

**DOI:** 10.1186/s13023-016-0400-8

**Published:** 2016-02-16

**Authors:** Andelko Hrzenjak

**Affiliations:** Department of Internal Medicine, Division of Pulmonology, Medical University of Graz, Auenbruggerplatz 15, A-8036 Graz, Austria; Ludwig Boltzmann Institute for Lung Vascular Research, Medical University of Graz, Graz, Austria

**Keywords:** Uterine malignancy, Gene fusion, Chromosomal translocation, *JAZF1/SUZ12*, *YWHAE/NUTM2A/B*

## Abstract

Endometrial stromal sarcomas (ESSs) belong to the rarest uterine malignancies (prevalence category <1-9/1,000,000). According to the new 2014 World Health Organisation (WHO) classification, they are separated into four categories; benign endometrial stromal nodules (ESNs), low grade endometrial stromal sarcomas (LG-ESSs), high-grade endometrial stromal sarcomas (HG-ESSs) and undifferentiated uterine sarcomas (UUSs). Due to heterogeneous histopathologic appearance these tumors still represent diagnostic challenge, even for experienced pathologists. ESSs are genetically very heterogeneous and several chromosomal translocations and gene fusions have so far been identified in these malignancies. To date the *JAZF1/SUZ12* gene fusion is by far the most frequent and seems to be the cytogenetic hallmark of ESN and LG-ESS. Based on present literature data this gene fusion is present in approximately 75 % of ESN, 50 % of LG-ESS and 15 % of HG-ESS cases. The frequency of *JAZF1/SUZ12* appearance varies between classic ESS and different morphologic variants. This gene fusion is suggested to become a specific diagnostic tool, especially in difficult borderline cases. In combination with the recently described *YWHAE/FAM22* gene fusion the *JAZF1/SUZ12* fusion could be used to differentiate between LG-ESS and HG-ESS. The purpose of this review is to summarize literature data published in last two and a half decades about this gene fusion, as a contribution to our understanding of ESS genetics and pathogenesis.

## Background

The pattern of different genetic rearrangements described in endometrial stromal sarcomas (ESSs) seems to be non-random. The chromosome arms 6p and 7p are frequently involved. One of the most common genetic alterations described in ESSs is the t(7;17)(p15;q21) chromosomal translocation. In some early studies this chromosomal change was described as a specific, non-random chromosome abnormality associated with low-grade endometrial stromal sarcomas (LG-ESSs) [1]. The first description of a recurrent chromosomal translocation was t(7;17)(p15;q21) in 1991 by Sreekantaiah et al., in a 58 years old women with metastatic LG-ESS [2]. At the chromosome breakpoints sites 7p15 and 17q21, fusion of two zinc-finger proteins was described; the so-called *JAZF1/JJAZ1* gene fusion [3]. Based on their structural features, these genes were given the acronyms *JAZF1*, for Juxtaposed with Another Zinc Finger gene, and *JJAZ1*, for Joined to *JAZF1* (lately named and referred in this review as *SUZ12* - suppressor of zeste-12 protein). This chromosomal translocation results in a chimeric RNA in which 5′ end (the first three exons from five in total) of the *JAZF1* gene on chromosome 7 is fused with the 3′ end (the last 15 exons from 16 in total) of the *SUZ12* gene on chromosome 17. During the past two and a half decades the classification of ESSs was modified several times, the most recent in 2014 [4, 5]. Thus, the *JAZF1/SUZ12* gene fusion should be revised with respect to the newest nomenclature.

## Detection methods

The most frequently used method for detection of *JAZF1/SUZ12* (formerly *JAZF1/JJAZ1*) gene fusion is reverse-transcriptase polymerase chain reaction (RT-PCR). However, one serious pitfall in detecting this gene fusion by RT-PCR is the preservation of the material used for RNA isolation. The cryo-preserved tissue is more suitable for isolation of high-quality RNA than formalin-fixed, paraffin-embedded (FFPE) tissue. The RT-PCR reaction in samples with poor-quality RNA may deliver false negative results. Huang et al. described four of ten frozen samples and only one of five FFPE ESS samples to be positive for *JAZF1/SUZ12* fusion transcripts [6]. In our study from 2005 we analysed 22 endometrial stromal tumors (ESTs); 20 LG-ESSs and 2 undifferentiated endometrial sarcomas (UESs) [7]. Out of 20 LG-ESSs, 16 (80 %) were positive for *JAZF1/SUZ12* gene fusion. We also compared PCR results performed with RNA isolated from both, cryo-preserved and FFPE tissue samples from three patients. Whereas the cryo-preserved material of two ESS cases was positive for *JAZF1/SUZ12* gene fusion, these cases were negative in their FFPE counterparts. Therefore we optimized an RT-PCR reaction for final PCR-product size of 93 bases of the *JAZF1/SUZ12* gene fusion sequence. Accordingly, two false-negative FFPE cases showed positive amplification of the specific *JAZF1/SUZ12* fusion sequence, which was confirmed by direct sequencing of the PCR product [7].

Nucci et al. were able to analyse only 16 of 24 LG-ESSs cases due to inadequate tissue quality and/or failed isolation of high-quality DNA [8]. Kurihara et al. collected 31 ESS cases, but could only analyse the *JAZF1/SUZ12* gene fusion in 18 of them, describing the rest as “noninformative” [9]. This was because the expression level of the reference gene (GAPDH) was lower than the cut-off level and suggests most probably the inadequate quality of the RNA from the FFPE material. The summary of the data published so far indicates that only approx. 75 % of all EST cases can be assessed for *JAZF1/SUZ12* fusion transcripts (Table 1, column “informative for *JAZF1/SUZ12*”).

**Table 1:** Incidence of *JAZF1/SUZ12* fusion transcript in uterine ESTs

First author (year)	ESN +/total (%)	LG-ESS +/total (%)	UES +/total (%)	Normal endometrium +/total (%)	Total Nr. of analyzed ESTs	Informative for *JAZF1/SUZ12* (%)
Koontz (2001) [3]	3/3 (100)	5/5 (100)	3/7 (43)	0/10 (0)	15	15 (100)
Micci (2003) [18]	-	1/4 (25)	-	-	4	4 (100)
Huang (2004) [6]	2/2 (100)	3/13 (23)	-	-	15	15 (100)
Hrzenjak (2005) [7]	-	16/20 (80)	0/2 (0)	0/10 (0)	22	22 (100)
Micci (2006) [36]	-	0/3 (0)	-	-	3	3 (100)
Oliva (2007) [10]	3/6 (50)	0/1 (0)	-	-	10	6 (60)
Nucci (2007) [8]	4/4 (100)	8/16 (50)	-	-	32	20 (63)
Kurihara (2008, 2010) [9, 12]	4/6 (67)	5/11 (45)	1/6 (17)	-	37	23 (62)
Li (2007) [33]	4/4 (100)	-	-	-	4	4 (100)
D'Angelo (2013) [37]	1/3 (33)	1/13 (8)	0/7 (0)	-	23	22 (96)
Isphording (2013) [45]	-	7/16 (44)	0/6 (0)	-	22	22 (100)
Total (%)	21/28 (75)	46/102 (45)	4/28 (14)	0/20 (0)	187	156 (83)

Abbreviations: *ESN* endometrial stromal nodule, *LG-ESS* low-grade endometrial stromal sarcoma, *UES* undifferentiated endometrial sarcoma, *+* positive for *JAZF1/SUZ12* gene fusion; total, number of all analyzed samples

In addition to RT-PCR, the interphase fluorescence in situ hybridisation (FISH) seems to be the method of choice for detection of the *JAZF1/SUZ12* fusion. The optimisation of the interphase FISH-based detection of *JAZF1/SUZ12* gene fusion in archival FFPE material was described by Oliva et al*.* [10]. The application of this method is of great importance because the majority of archival material is available in this form, whereas cryo-preserved tissue is rare. However, some potential pitfalls of the FISH analysis must be taken into account: i) false monosomy/polysomy can occur due to sectioning artifacts, ii) the FISH technology is quite time-consuming and labor-intensive. In order to exclude false-negative results on archival FFPE tissue samples, further optimisation of accurate RT-PCR method and the combination of both methods, RT-PCR and FISH, is highly recommended.

## Frequency in different EST variants

Studies concerning the *JAZF1/SUZ12* gene fusion in different ESS variants are rare. Based on histological appearance, the accurate comparison of different variants of all described ESS cases is challenging because; (i) criteria for histological classification based on morphology varied in different studies and institutions, and/or they were not precisely specified, (ii) the number of cases in different categories was usually too small to make a precise conclusion on the frequency of *JAZF1/SUZ12* fusion in ESSs of different histologic subtypes.

One comparative genomic hybridisation (CGH) study showed that chromosomal aberrations in ESTs are heterogeneous and do not correlate to histologic grades [11]. Huang et al. described the presence of the *JAZF1/SUZ12* fusion in one case of endometrial stromal nodule (ESN) with smooth muscle (SM) differentiation [6]. In their studies Kurihara et al. found the *JAZF1/SUZ12* fusion transcript in 4 out of 6 (67 %) ESNs, 5 out of 12 (45 %) LG-ESSs and in 1 out of 3 (33 %) UESs with nuclear uniformity (UES-U), whereas all three UES cases with nuclear pleomorphism (UES-P) were negative [9, 12]. These data for undifferentiated tumors indicate the heterogeneity of this category, which now according to the 2014 WHO classification is separated into high-grade endometrial stromal sarcomas (HG-ESSs) and undifferentiated uterine sarcomas (UUSs).

Overall, published data suggest that the *JAZF1/SUZ12* fusion is present only in a subset of primary ESTs. These variations in prevalence might be based on (i) methods used for tissue collection and/or preservation, (ii) *JAZF1/SUZ12* detection methods (RT-PCR versus FISH), or (iii) differences between patient populations. In respect to the ethnic consideration, two studies were published since now describing clinicopathological features of uterine sarcomas in South African women [13] and molecular characterisation of a population-based series of ESSs in Kuwait [14]. In the second study the JAZF1 rearrangement was reported in 70 % of LG-ESSs, however the presence/absence of the *JAZF1/SUZ12* gene fusion was not clearly specified. Altogether these data indicate the heterogeneity of ESS/UES, an issue which can only be solved by analysing larger number of samples.

Data summarizing the incidence of *JAZF1/SUZ12* gene fusion are presented in Table 1. Oliva et al. studied a subset of 10 ESTs; 9 ESNs and one ESS with smooth muscle differentiation (SM). Six out of 10 cases were assessable for detection of the *JAZF1/SUZ12* fusion and fusion signals were detected in 3 cases (50 %), both in endometrial stromal and in SM components [10]. The authors state that these findings support the idea of a common origin for endometrial stromal and SM components in EST. Furthermore, they suggest this detection as a supportive method for diagnosis of ESTs with SM differentiation. This is of high significance because the distinction between well-differentiated SM neoplasms, such as cellular leiomyoma, and ESS with predominant SM differentiation can be challenging even for experienced pathologists.

Huang and co-workers categorized 15 ESSs based on their histological appearance and showed that the prevalence of *JAZF1/SUZ12* fusion was mainly limited to the subset of ESSs with classic histology [6]. Beside these cases with classic histology, they detected the *JAZF1/SUZ12* fusion in one ESN of mixed SM variant. In our study we have found 12 out of 14 classic LG-ESSs to be positive for *JAZF1/SUZ12* fusion. Two ESS variants, which included 1 mixed low-grade ESS with epithelioid (EP) and sex cord-like differentiation (SC) and 1 with a fibromyxoid (FM) feature were also positive, whereas the third ESS with SC differentiation was negative [7]. Both UUSs analysed in our study were negative for *JAZF1/SUZ12* fusion. This supports the hypothesis, that there is low, if any, pathologic relationship between LG-ESSs and undifferentiated uterine sarcomas. We also analyzed normal endometria (n = 10), leiomyomas (n = 5), leiomyosarcomas (n = 5), lung carcinomas (n = 3), gastric carcinomas (n = 3) and hepatic carcinomas (n = 3), all of which were negative for the *JAZF1/SUZ12* fusion transcript.

In their study with 24 cases of uterine tumors resembling ovarian sex cord tumors (UTROSCT), Staats et al. used both FISH and RT-PCR to detect the *JAZF1/SUZ12* fusion transcript, but none of 24 UTROSCT samples were positive [15]. These data indicate that UTROSCT are not related to ESTs and represent distinct entity. Kurihara et al. found the *JAZF1/SUZ12* fusion transcript in one of two ESNs with sex SC differentiation [12]. By using FISH, Nucci et al. detected two ESNs with smooth muscle differentiation to be positive for the *JAZF1/SUZ12* fusion [8]. The *JAZF1/SUZ12* fusion transcript was not only found in primary tumors, but also in metastases and in a primary extrauterine ESS [6]. Kurihara et al. reported about 24 ESTs assayed for *JAZF1/SUZ12* fusion transcript; 6 ESN, 12 ESS and 6 UES cases [9, 12]. Four out of 6 ESNs, six out of 12 ESSs and one UES-U were positive, whereas all 3 UES-P cases were negative for *JAZF1/SUZ12* gene fusion (Table 2). These data clearly indicate the heterogeneity of UESs and can be now, based on WHO 2014 classification distinguishing between HG-ESS and UUS, observed in a different context.

**Table 2:** Distribution of JAZF1/SUZ12 fusion transcript in different histological subtypes of ESTs

	ESN, total (+)			ESS, total (+)						UES, total (+)	
Ref.	Classic	SM	Mixed	Classic	SM	EP	FM	SC	Mixed	UES-U	UES-P
[3]	3^a^ (3)			5^a^ (5)							
[6]	1 (1)	1 (1)	-	3 (3)	2 (0)	2 (0)	2 (0)	-	-	-	-
[7]	-	-	-	16 (14)	-	-	1 (1)	1 (0)	1 (1)	-	2 (0)
[8]	2 (2)	2 (2)		16 (8)							
[9], [12]	6 (4)	-	-	11 (5)	-	-	-	1 (0)	-	3 (1)	3 (0)
[10]	5 (2)	-	-	1 (1)	-	-	-	-	-	-	-
[35]	2 (1)	-	1 (0)	5 (1)	1 (0)	-	-	4 (0)	3 (0)	3 (0)	4 (0)
[44]	-	-	-	16^a^ (7)	-	-	-	-	-	6^a^ (0)	-
Total Nr.	19 (13)	3 (3)	1 (0)	73 (44)	3 (0)	2 (0)	3 (1)	6 (0)	4 (1)	12 (1)	9 (0)
+ / total (*n* = 135)	9.6 %	2.2 %	-	32.6 %	-	-	0.7 %	-	0.7 %	0.7 %	-
+ / type	69.6 %			50.5 %						4.8	
+ / subtype	68 %	100 %	-	60 %	-	-	33 %	-	25 %	8 %	-

Abbreviations: *Ref* references, *ESN* endometrial stromal nodule, *LG-ESS* low-grade endometrial stromal sarcoma, *UES* undifferentiated endometrial sarcoma; classic, with classic histology, *SM* with smooth muscle differentiation, *EP* with epithelioid features, *FM* with fibromyxoid features, *SC* with sex cord-like differentiation, *U* with nuclear uniformity, *P* with nuclear pleomorphism

^a^,morphologic subtype was not precisely described in original paper; +, positive for *JAZF1/SUZ12* fusion transcript; total, number of samples suitable for analyses (cases described in original paper as “non informative” or “not analyzed” were excluded from calculations)

Primary extrauterine ESSs are very rare mesenchymal neoplasms with morphologic features similar to their uterine counterparts. Sato et al. confirmed the *JAZF1/SUZ12* gene fusion by RT-PCR and FISH analysis in one primary extrauterine ESS arising from the extraperitoneal portion of the round ligament in the inguinal canal [16]. Therefore, they suggested detection of *JAZF1/SUZ12* gene fusion as an additional tool in the diagnosis of extrauterine ESTs. The *JAZF1/SUZ12* fusion transcript has been analysed in six additional primary extrauterine LG-ESSs. One out of six samples was positive both by RT-PCR and by FISH, indicating lower prevalence of *JAZF1/SUZ12* gene fusion in extrauterine in comparison to uterine ESSs [17]. Few additional extrauterine ESSs were analysed sporadically [7, 18, 19] and, therefore, the significance of this analysis as a diagnostic tool for primary extrauterine ESTs is limited by both, the scarcity and by the low prevalence of these tumors.

Short summary of *JAZF1/SUZ12* gene transcript distribution in different histologic subtypes of ESTs is shown in Table 2. Albeit, this summary should be taken with caution as molecular diagnostic testing of ESTs for *JAZF1/SUZ12* gene fusion is still not a standard and histological classification of described ESTs is frequently not complete. Furthermore, different detection methods were used in different studies, which might have an influence on the final results. Altogether, these data suggest that *JAZF1/SUZ12* fusion is frequently but not consistently present in ESNs and in ESTs of classic histology and less often in other histological subtypes. These data support the idea about genetic heterogeneity between ESTs with different histology and might have an important implication on the diagnosis of different EST subtypes.

## Fusion protein - Putative biological functions

The ESTs exhibit different patterns of genetic aberrations, but rearrangements of the chromosomes 6, 7 and 17 seem to be the most common. At the 7p15 and 17q21 breakpoints genes named *JAZF1* and *SUZ12* (formerly *JJAZ1*) were identified. It has been shown that they are associated with the t(7;17)(p15;q21) alteration in ESSs [3]. In cDNA sequences from both genes there are so-called zing finger motifs, which are characteristic for DNA-binding proteins [20, 21]. In our previous study, we showed that the breakpoint in the *JAZF1/SUZ12* fusion sequence, detected by direct sequencing of positive PCR-products, was the same in all 16 positive cases [7]. In this break-point G-435 from the *JAZF1* sequence was followed by A-468 from the *SUZ12* sequence and was, therefore, identical with the break-point described by other authors in a limited number of cases [3, 6, 18]. These findings indicate that the break-point in the fusion gene sequence is highly conserved.

As shown by Northern blot hybridization and RNase protection assay, the cDNAs for *JAZF1* and *SUZ12* represent transcripts for predicted proteins composed of 243 and 739 amino acids, respectively [3]. Tumor-specific mRNA transcript for predicted protein contains 5-prime end of the *JAZF1* and 3-prime end of the *SUZ12* sequence, with retaining zinc finger motifs from both genes. Two zinc finger domains in *JAZF1* gene show homology to the yeast protein Sfp1p, which negatively regulates the G2/M transition in *Streptomyces cerevisiae* by transcriptional activation of some other genes like *PDS1* and *Esp1p* [3]. Based on this, the authors suggested that the *JAZF1/SUZ12* chimeric protein might disrupt transcription in lineage-specific manner. The loss of expression of a normal *JAZF1* has been shown in some ESS cases [3]. Additionally, a growing body of evidence is showing that the loss of expression for normal version of the *JAZF1* in various malignancies, thus suggesting that the *JAZF1* may have a role as a tumor suppressor gene [22]. Though, the specific function of the *JAZF1* and *SUZ12* genes in human malignant cells is still not known.

Using *JJAZ1*-specific antibody, Li et al. showed for the first time that *JAZF1/SUZ12* RNA is translated into the fusion protein in immortalized, normal human endometrial stromal cells (HESC) [23]. However, these findings are not consistent with data published latter by Panagopoulos et al., who did not find this chimeric transcript in the same cell line. Few possible explanations were discussed, including different group of examined cells, different culturing conditions and differences in the PCR approach [24]. Furthermore, it might be that the *JAZF1/SUZ12* fusion RNA in normal endometrial stroma is not a product of the gene fusion, but rather of some kind of RNA trans-splicing with unknown mechanisms.

The highest *JAZF1* expression level was detected in adult testis, moderate in placenta, colon, prostate and ovary [25]. Recently, high expression level was detected in liver and adipose tissue, as well as in induced preadipocytes, suggesting its potential role in lipid metabolism [26, 27]. It has been shown that *JAZF1* encodes a transcriptional corepressor which physically binds to the orphan nuclear receptor TAK1, which is a regulator of transcription [25]. Interestingly, TAK1 receptor deficient mice are protected against obesity-linked inflammation, hepatic steatosis, and insulin resistance [28]. However, its potential role in malignant diseases still remains obscure.

The wild-type JAZF1 protein is expressed in normal endometrium. Thus, it has been suggested that the fusion protein might disrupt transcription in a lineage-specific manner [3]. The *JAZF1* shows sequence similarities to the yeast protein Sfp1p, which activates the gene *PDS1* and inhibits the transition from G2 phase to mitosis in *Saccharomyces cerevisiae*. However, in transfected human cells the JAZF1 protein does not have any effect on this transition [3]. Single nuclear polymorphisms in the *JAZF1* introns have been reported to be associated with an increases risk of type 2 diabetes [29] and with increased susceptibility to prostate cancer [30].

The *JJAZ1* orthologue gene in *Drosophila* is mutated in flies with the *SuZ12* phenotype [31]. The SuZ12 protein is an essential component of the polycomb repression complex 2 (PRC2), which plays a role in methylation of lysines 9 and 27 of histone 3. This leads to chromatin remodelling, increased chromatin compactness and transcriptional gene silencing [32]. Introduction of the *JAZF1/SUZ12* expression vector into cultured HEK-293 (human embryonic kidney) cells significantly promoted the proliferation of these cells and inhibition of apoptosis [33]. This effect was dependent on suppression of the endogenous *SUZ12* and was present only in cultured cells with silenced endogenous *SUZ12* alleles. In addition, expression levels of EZH2 protein and total trimethyl histone 3 lysine 27 (H3K27), which are reduced when *SUZ12* is deficient in these cells, were restored to normal levels. Some published data indicate that *SUZ12* is important for stabilization of the EZH2 protein [34, 35]. Furthermore, the expression of the *JAZF1/SUZ12* protected HEK-293 cells from serum deprivation and from hypoxia-induced apoptosis. Based on their results, Li et al. suppose that the *SUZ12* might have features of both, an oncogene and a tumor suppressor gene [33]. Relatively high percentage of positive ESSs, as well as highly conserved breakpoint in the fusion gene sequence, suggest that this translocation might play an important role in ESS/UES pathogenesis. Since this gene fusion is present also in ESNs, the possibility exists that malignant ESTs can develop from this benign form. However, the implication of *JAZF1/SUZ12* gene fusion for the biology of ESTs is not clarified yet.

## Other chromosomal rearrangements in ESSs

Another chromosomal rearrangement has been reported in the ESSs, containing the *JAZF1* gene and the gene sequence for so called PHD finger protein 1 (*PHF1*), located at chromosomal band 6p21 [36]. The *PHF1* gene shows high sequence homology to the *Drosophila* polycomblike (*PCL*) gene and encodes a protein with two zinc finger motifs. This new *JAZF1/PHF1* fusion gene was first described in two of three analysed ESSs. D'Angelo and colleagues described a correlation between the occurrence of PHF1 rearrangement and ESSs with sex cord differentiation [37]. In their study all 7 cases with sex cord differentiation were positive for PHF1 rearrangement, whereas only one was positive for JAZF1 rearrangement. One recent study, with a total of 40 cases, showed that 36 % of LG-ESSs (8 of 22) and none of 17 UESs (10 with nuclear uniformity and 7 with nuclear pleomorphism) were positive for the JAZF1/PHF1 rearrangement [38].

Panagopoulos et al. described an endometrial sarcoma cell line, the JHU-ESS1, containing the *JAZF1/PHF1* gene fusion [39]. They confirmed by direct sequencing that exon 3 of *JAZF1* (nucleotide 626) was fused with exon 2 of *PHF1* (nucleotide 276). Thus, this fusion differ from two chimeras previously described by Micci et al., where exon 2 or 3 of *JAZF1* was fused with exon 1 or 2 of *PHF1*, respectively [36]. At the junction in JHU-ESS1 cells, an insertion of 26 nucleotides from intron 3 of *JAZF1* (position 96834–96859) was found, thus maintaining an open reading frame of the predicted chimeric transcript. The open reading frame of this fusion consists of 130 amino acids (aa) from *JAZF1* and 7 additional aa from an intronic sequence fused with the proline at position 21 in *PHF1*. Thus, the putative fusion protein would contain 684 aa. Micci et al. showed that the *JAZF1/PHF1* fusion sequence has an open reading frame coding for 657 amino acid residues in one ESS case and 727 amino acid residues in the second ESS case. Both putative proteins retain one zinc finger domain from *JAZF1* and two zinc finger domains from *PHF1* gene and are under control of the *JAZF1* promoter [36]. Although the specific functions of *JAZF1* and the *PHF1* and their role in ESS-pathogenesis are not clear, one can expect that based on their zinc finger motifs they are involved in regulation of transcriptional processes in tumor cells. Whereas all other gene fusions from ESTs were detected exclusively in these tumors, the *JAZF1/PHF1* fusion was recently described in the sarcoma of the heart, thus being the only one detected outside of the EST spectrum [40].

Recently, a new chromosomal translocation t(10;17) (q22;p13) was reported in a distinct group of ESS, fusing two genes: *YWHAE*, encoding a member of the 14-3-3 family, and either *NUTM2A* or *NUTM2B* (formerly reported as *FAM22A* or *FAM22B*), respectively [41, 42]. Based on present studies, tumors with *YWHAE/NUTM2* rearrangements are associated with high-grade morphology and aggressive clinical behaviour [41, 43–47]. However, they usually do not behave as aggressively as UUS, the most aggressive form of ESTs. The *YWHAE/NUTM2* and the *JAZF1/SUZ12* fusions seem to be mutually exclusive. Therefore, both may be considered as a support to morphology-based diagnosis, especially in difficult borderline cases. ESSs with *JAZF1* and *YWHAE/NUTM2* rearrangements also differ concerning the estrogen- and progesterone-receptor expression, the *YWHAE/NUTM2* tumors being consistently negative [48]. Although the impact factor of these two fusions on prognostic and possible therapeutic implications must be further investigated, these findings already suggest that application of the hormonal therapy in the *YWHAE/NUTM2* positive ESSs may be less meaningful.

In 2014, Dewaele et al. detected a novel gene fusion *MBTD1-CXorf67* in a subset of LG-ESSs [49]. However, detection of putative fusion protein and plausible mechanistic explanation requires future studies. Two novel gene fusions, *MEAF6/PHF1* and *ZC3H7B/BCOR*, were detected in a subset of ESSs with t(1;6)(p34;p21) and der(22)t(X;22)(p11;q13) genetic alterations, respectively [50–52]. It is to be expected that a comprehensive genome and transcriptome analyses, as performed very recently by Choi et al, [53] will help us detecting new genetic alterations in different EST variants.

## Conclusions and future perspectives

ESSs are genetically heterogeneous malignancies, with following chromosomal translocations identified: t(7;17)(p15;q21), t(6;7)(p21;p15), t(6;10;10)(p21;q22;p11), t(1;6)(p34;p21) and t(10;17)(q22;p13). They result in following gene fusions: *JAZF1/SUZ12*, *PHF1/JAZF1*, *EPC1/PHF1*, *MEAF6/PHF1* and *YWHAE/FAM22*. Data published so far show that the *JAZF1/SUZ12* fusion is a genetic hallmark of ESNs and of LG-ESSs. Presence of this fusion in ESNs might suggest that: (i) this genetic alteration is an early event in the tumor development, and (ii) ESN is a benign precursor of the malignant LG-ESS. This gene fusion is present in 75 % of ESNs, 45 % of LG-ESSs and in approx. 14 % of HG-ESSs and the frequency of appearance varies between classic ESS and different morphologic variants. A growing community of authors suggests that the *JAZF1/SUZ12* gene fusion should become a specific diagnostic tool, especially when the diagnosis of ESSs is unclear or difficult due to various reasons. It might be particularly useful for differential diagnosis between LG-ESSs and smooth muscle tumors of the uterus, which are always negative for that fusion. The fact that *JAZF1/SUZ12* gene fusion is only sporadically positive in HG-ESSs and UUSs support the hypothesis, that these tumors bear no pathological relationship with LG-ESSs.

Novel chromosomal translocation (t(10;17)) might also be useful as a differential diagnostic tool between LG-ESSs and HG-ESSs. Since the prognosis and the 5-year survival rate of LG-ESSs and HG-ESSs are drastically different, the precise distinction between these entities is clinically very important. Although it is very challenging to foresee the whole potential benefit behind these new findings, further investigations in this field will certainly provide novel insights into our understanding of ESSs pathogenesis.

Elucidation of the functions of fusion genes/proteins in uterine sarcomas will further improve our understanding of the oncogenic process in these malignancies. This might lead to the identification of new therapeutic targets for the treatment of uterine sarcoma.

## Abbreviations

BCOR: BCL6 corepressor
CXofr67: chromosome X open reading frame 67
ESN: endometrial stromal nodule
ESS: endometrial stromal sarcoma
ESS-EP: endometrial stromal sarcoma with epithelioid differentiation
ESS-FM: endometrial stromal sarcoma with fibromyxoid feature
ESS-SC: endometrial stromal sarcoma with sex cord-like differentiation
ESS-SM: endometrial stromal sarcoma with smooth muscle differentiation
EST: endometrial stromal tumor
EZH2: enhancer of zeste 2 polycomb repressive complex 2 subunit
FFPE: formalin-fixed, paraffin-embedded
FISH: fluorescence *in situ* hybridisation
HESC: human endometrial stromal cells
HG-ESS: high-grade endometrial stromal sarcoma
JAZF1: juxtaposed with Another Zinc Finger gene
JJAZ1: joined to *JAZF1*
LG-ESS: low-grade endometrial stromal sarcoma
MBTD1: mbt domain containing 1
MEAF6: MYST/Esa1-associated factor 6
NUTM2A: NUT family member 2A
NUTM2B: NUT family member 2B
PCL: polycomblike
PHF1: PHD finger protein 1
PRC2: polycomb repression complex 2
SUZ12: suppressor of zeste-12 protein
UES-P: undifferentiated endometrial sarcoma with nuclear pleomorphism
UES-U: undifferentiated endometrial sarcoma with nuclear uniformity
UTROSCT: uterine tumor resembling ovarian sex cord tumor
UUS: undifferentiated uterine sarcoma
YWHAE: tyrosine 3-monooxygenase/tryptophan 5-monooxygenase activation protein, epsilon
ZC3H7B: zinc finger CCCH-type containing 7B
